# Screening Performance of Edmonton Symptom Assessment System in Kidney Transplant Recipients

**DOI:** 10.3390/jcm9040995

**Published:** 2020-04-02

**Authors:** Yuri Battaglia, Luigi Zerbinati, Giulia Piazza, Elena Martino, Michele Provenzano, Pasquale Esposito, Sara Massarenti, Michele Andreucci, Alda Storari, Luigi Grassi

**Affiliations:** 1 Nephrology and Dialysis Unit, St. Anna University Hospital, 44124 Ferrara, Italy; a.storari@ospfe.it; 2 Institute of Psychiatry, Department of Biomedical and Specialty Surgical Sciences, University of Ferrara, 44124 Ferrara, Italy; zrblgu@unife.it (L.Z.); pzzgli@unife.it (G.P.); mrtlne1@unife.it (E.M.); msssra@unife.it (S.M.); luigi.grassi@unife.it (L.G.); 3 Nephrology and Dialysis Unit, Department of Health Sciences, Magna Graecia University, 88100 Catanzaro, Italy; michiprov@hotmail.it (M.P.); andreucci@unicz.it (M.A.); 4 Department of Internal Medicine, Division of Nephrology, Dialysis and Transplantation, University of Genoa and IRCCS Ospedale Policlinico San Martino, 16132 Genoa, Italy; pasqualeesposito@hotmail.com

**Keywords:** ESAS, psychiatric morbidity, distress, kidney transplantation

## Abstract

An average prevalence of 35% for psychiatric comorbidity has been reported in kidney transplant recipients (KTRs) and an even higher prevalence of other psychosocial syndromes, as defined by the Diagnostic Criteria for Psychosomatic Research (DCPR), has also been found in this population. Consequently, an easy, simple, rapid psychiatric tool is needed to measure physical and psychological symptoms of distress in KTRs. Recently, the Edmonton Symptom Assessment System (ESAS), a pragmatic patient-centred symptom assessment tool, was validated in a single cohort of KTRs. The aims of this study were: to test the screening performances of ESAS for the International Classification of Diseases-10th Revision (ICD-10) psychiatric diagnoses in KTRs; to investigate the optimal cut-off points for ESAS physical, psychological and global subscales in detecting ICD-10 psychiatric diagnoses; and to compare ESAS scores among KTR with ICD-10 diagnosis and DCPR diagnosis. 134 KTRs were evaluated and administered the MINI International Neuropsychiatric Interview 6.0 and the DCPR Interview. The ESAS and Canadian Problem Checklist (CPC) were given as self-report instruments to be filled in and were used to examine the severity of physical and psychological symptoms and daily-life problems. The physical distress sub-score (ESAS-PHYS), psychological distress sub-score (ESAS-PSY) and global distress score (ESAS-TOT) were obtained by summing up scores of six physical symptoms, four psychological symptoms and all single ESAS symptoms, respectively. Routine biochemistry, immunosuppressive agents, socio-demographic and clinical data were collected. Receiving Operating Characteristic (ROC) analysis was used to examine the ability of the ESAS emotional distress (DT) item, ESAS-TOT, ESAS-PSY and ESAS-PHYS, to detect psychiatric cases defined by using MINI6.0. The area under the ROC curve for ESAS-TOT, ESAS-PHYS, ESAS-PSY and DT item were 0.85, 0.73, 0.89, and 0.77, respectively. The DT item, ESAS-TOT and ESAS-PSY optimal cut-off points were ≥4 (sensitivity 0.74, specificity 0.73), ≥20 (sensitivity 0.85, specificity 0.74) and ≥12 (sensitivity 0.85, specificity 0.80), respectively. No valid ESAS-PHYS cut-off was found (sensitivity <0.7, specificity <0.7). Thirty-nine (84.8%) KTRs with ICD-10 diagnosis did exceed both ESAS-TOT and ESAS-PSY cut-offs. Higher scores on the ESAS symptoms (except shortness of breath and lack of appetite) and on the CPC problems were found for ICD-10 cases and DCRP cases than for ICD-10 no-cases and DCPR no-cases. This study shows that ESAS had an optimal screening performance (84.8%) to identify ICD-10 psychiatric diagnosis, evaluated with MINI; furthermore, ESAS-TOT and ESAS-PSY cut-off points could provide a guide for clinical symptom management in KTRs.

## 1. Introduction

Kidney transplantation (KT) is the most cost-effective modality of renal replacement therapy for patients with stage 5 chronic kidney disease [1]. KT is a stressful condition with the involvement of complex and bidirectional mechanisms including biological and psychosocial factors [2] and requiring a process of significant bio-psycho-social adaptation.

Even though KT is associated with improved clinical outcomes and better quality of life compared with dialysis [3,4], the risk of psychiatric and psychosocial comorbidity in kidney transplant recipients (KTRs) has been shown to be high, with rates varying according to the different measures, psychometric tools, standardized interviews, and international classification systems such as the Diagnostic and Statistical Manual of Mental Disorders (DSM) or the International Classification of Diseases (ICD). Mood, anxiety, and stress-related disorders have been the most common psychiatric conditions observed in 25% to 40% of KTRs in the post-transplant period [5,6,7,8,9,10,11]. These disorders are associated with potential co-morbidity and unfavorable outcomes such as functional impairment, reduced adherence to medical care [12,13], decreased quality of life, increased health costs [14,15], and higher mortality [16]. 

Besides traditional psychiatric diagnoses, however, a wide array of other psychological dimensions that can be only partially explained by the DSM and ICD diagnostic models [17,18] have been more recently reported in KTRs. With respect to this, the Diagnostic Criteria for Psychosomatic Research (DCPR) were used as a complementary integration to the traditional nosographic psychiatric criteria for a better understanding of psychological distress in KTRs. In fact, the occurrence of DCPR diagnoses was found to be almost double than the ICD psychiatric diagnoses—especially abnormal illness behaviour, irritability, alexithymia, somatisation, and demoralization [19]. The high prevalence rate of psychiatric disorders and relevant psychosocial conditions supported the need to use a tool to assess physical and psychological symptoms of distress in KTRs. The urgency for standardized psychiatric and psychosocial screening for distress in KTRs is also supported by the high prevalence of alexithymia [20], defined as a difficulty in identifying and describing emotions. The most commonly used psychiatric screening tools are however time-consuming and difficult for patients to complete [21,22,23,24,25,26,27]. A pragmatic patient-centred symptom assessment tool with visual analogue scale, the Edmonton Symptom Assessment System (ESAS), has been validated in dialysis patients [28] and, recently, in a single kidney-transplant cohort [29,30,31]. ESAS scores varies with the burden and the effects of disease and is correlated with all domains of health-related quality of life in dialysis patients. ESAS is also useful for the longitudinal assessment of physical and psychological symptom burden in end-stage renal disease; it is also a practical tool that allows the nephrologist to monitor the burden of physical and psychological symptoms of distress over time, allowing for an easy reassessment. The simplicity of the ESAS allows for its easy integration into routine clinical practice [32].

Since post-transplant patients are less likely to return to the nephrologist after the first-year than during the first year, vis-à-vis screening for mental disorders is less feasible, and an easy tool for mental health screening, which can be delivered by a non-doctor, is therefore needed [33]. Additionally, the psychosocial challenges that recipients face after the first-year post transplantation are significantly different from those they face during their first year [34].

Against this background, the primary aim of this study is to test the screening performances of the ESAS for the ICD10th Revision (ICD-10) psychiatric diagnoses in KTRs after their first-year post transplantation. The secondary aim is to investigate the optimal cut off points for ESAS physical, psychological, and global subscales in detecting ICD-10 psychiatric diagnoses. Thirdly, we compared the ESAS scores in KTRs who received an ICD-10 diagnosis, with those who received a DCPR diagnosis and with those who did not receive any diagnosis.

## 2. Materials and Methods

This study was carried out in prevalent KTRs who were followed up in a Nephrology Unit. Inclusion criteria were moderate/high ability of KTRs to perform ordinary activities (Karnofsky Performance Status score ≥50) [35], absence of cognitive disorders (Mini Mental State Examination ≥24) and having received the graft after one year or more. Each patient was informed about the aims of the study, with ethical approval of the study obtained from the Hospital Ethics Committee for Human Research (approval code is 151297, 17 March, 2016).

Participants meeting the inclusion criteria were approached during one of their routine follow up nephrological visit during an index week and were met by the same psychiatrist of the Consultation-Liaison Psychiatric Service, University Psychiatry Unit of the same Hospital, with an extensive experience in psychosomatic research. 

Each patient was individually administered with the MINI International Neuropsychiatric Interview (MINI6.0) and the Diagnostic Criteria for Psychosomatic Research (DCPR) interview. The two interviews took about two hours. Before the diagnostic interviews, the Edmonton Symptom Assessment System (ESAS) and Canadian Problem Checklist (CPC) were given as self-report tools to be filled in.

The MINI6.0 [36] is a short, structured diagnostic interview that has been validated against both the Structured Clinical Interview for DSM diagnoses (SCID-P) and the Composite International Diagnostic Interview (CIDI) for ICD-10 diagnoses in different countries, including Italy [37,38]. In this study, we followed the ICD-10 classification to make a psychiatric diagnosis. 

The DCPR interview [39] investigates a set of 12 syndromes organized in 4 different clusters, namely abnormal illness behaviour (AIB) (e.g., disease phobia, thanatophobia, health anxiety, illness denial), somatization and its different expressions, irritability and other relevant clinical constructs, including demoralization and alexithymia. The DCPR has been extensively used in medical settings and it enables clinicians to identify psychosocial conditions to a much greater extent than the DSM or ICD classification by providing information on specific psychological and psychosocial factors affecting a prevalent number of patients suffering from a given group of medical illnesses [40]. Following other authors’ recommendations, [6,41] we considered those patients who resulted positive to one or more DCPR diagnosis as “cases”, for the purpose of this study. 

The ESAS [42] was used to examine the severity of physical (i.e., pain, tiredness, nausea, drowsiness, lack of appetite, shortness of breath) and psychological symptoms (i.e., depression, anxiety, feeling of not being well) on a 0 (no symptom) to 10 (the worst symptom) scale. In agreement with Howell and Olsen [43], an optional tenth psychological symptom was also added in this study, specifically the emotional distress item which corresponds to the Distress Thermometer (DT), a worldwide validated tool to measure distress also on a 0–10 VAS that has been validated in a range of chronic disease populations, including the renal population [44]. The physical distress sub-score (ESAS-PHYS) is the sum of scores for the six physical symptoms, while the psychological distress sub-score (ESAS-PSY) is the sum of the scores of the four psychological symptoms. A Global ESAS-TOT score is given by summing up all the scores on the single ESAS symptoms. The ESAS has been largely used in palliative care and in medically ill patients, including renal haemodialysis and kidney transplant patients, showing good psychometric properties [8,45]. 

The CPC was used to assess possible problems the patient has to deal with [46]. The CPC consists of a list of 21 problems each rated in a yes/no (0–1) format and grouped into 6 categories (practical, social/family, emotional, spiritual, informational, and physical problems). The Italian versions of the ESAS and the DT, which have been shown to have good levels of validity, were used in this study [47,48,49,50].

Socio-demographic and clinical data, including immunosuppressive agents, were collected, and routine biochemistry was repeated. Kidney function was evaluated with the estimated glomerular filtration rate (eGFR) according to the equation from the Modification of Diet in Renal Disease Study (MDRD) [51] and routine biochemistry was determined using standard auto analyser techniques.

### Statistical Analysis

Statistical analysis was performed using SPSS-22 package. Procedures included descriptive statistics to examine the general characteristics of the sample. Continuous variables were reported as either mean ± standard deviation (SD) or median and interquartile range (IQR) based on their distribution. Comparison among ICD-10 or DCPR categories was assessed by unpaired t-test. Two sided, known variance, test was computed. Comparisons among the combined ICD-10 plus DCPR categories were tested by means of one-way Analysis of Variance (ANOVA). Categorical variables were showed as frequencies (%) and analysed using Chi-square test.

Receiving Operating Characteristic (ROC) analysis was used to explore the optimal DS cut-off score in detecting clinical cases in our sample, specifically to examine the ability of the ESAS DT item, ESAS-TOT, ESAS-PSY and ESAS PHYS, to detect psychiatric cases defined by using MINI6.0 (caseness). Combined sensitivities and specificities were visualized in the ROC curve. The Area Under the Curve (AUC) provides an estimate of overall discrimination for evaluations of possible adequate cut off values of the ESAS. AUC of 0.5–0.7 indicates low accuracy, 0.7–0.9 indicates moderate accuracy and 0.9–1.0 indicates high accuracy [41]. The Youden index (J) was computed to find a cut-point that maximizes the variable’s differentiating ability from the ROC curves. J is defined as the variable’s value for which equal weight is given to sensitivity and specificity [52]. 

## 3. Results

### 3.1. Characteristics of the Sample

Data pertaining to 134 out of 143 consecutive outpatients were collected (9 patients declined to participate: 6 for work or family reasons, and 3 because of health reasons), of whom most were males (67.2%). The mean age was 56.13 ± 12.0 years (Table 1). Forty-one (30.6%) patients reported previous psychological disorders, of which adjustment disorders (28.5%) were the most prevalent diagnosis. The median time post-transplant was 85 (IQR = 34.75–178.5).

Of the total sample, 46 patients (34.3%) met the criteria for an ICD-10 diagnosis (ICD-10 cases), specifically reaction to severe stress and adjustment disorders (*n* = 21, 15.7%), anxiety disorders (*n* = 14, 10.4%), and mood disorders (*n* = 11, 8.2%: major depression *n* = 3, 2.2% and other mood disorders *n* = 8, 6%).

Regarding DCPR, 85 (63.4%) had at least 1 DCPR diagnosis (of whom 43 had 1 diagnosis and 42 more than one diagnosis). The specific characteristics and the details about the DCPR syndromes are reported elsewhere [19].

### 3.2. Screening Performance and Optimal Cut-Off Points of the ESAS for Detecting ICD-10 Diagnoses

**Figure 1:**
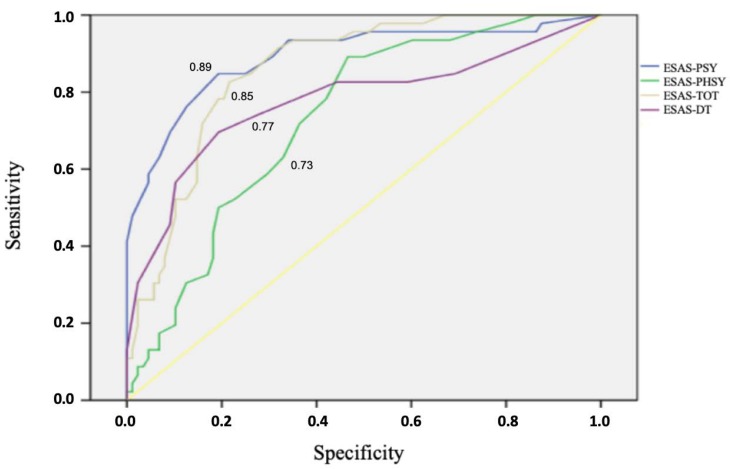
Receiver operating characteristic analysis of caseness on MINI is illustrated. ESAS: Edmonton Symptom Assessment System; ESAS-DT: distress Item; ESAS-PHYS: physical distress; ESAS-PSY: psychological distress; ESAS-TOT: global distress; MINI: Mini-International Neuropsychiatric Interview.

The area under the ROC curves for ESAS-TOT, ESAS-PHYS, ESAS-PSY and DT item, were 0.85 (95%CI = 0.79–0.92), 0.73 (95%CI = 0.64–0.81), 0.89 (95%CI = 0.82–0.95), and 0.77 (95%CI = 0.68–0.86), respectively (Figure 1). The ROC curves showed relatively moderate accuracies of psychological distress item, ESAS-TOT and ESAS-PSY, to detect caseness.

The psychological distress cut-off point of ≥4 provides the balance between sensitivity of 0.74 and specificity of 0.73 for caseness, maximizing sensitivity at cost of specificity. Similarly, the ESAS-TOT and ESAS-PSY optimal cut-off points were 20 (sensitivity 0.85, specificity 0.74) and 12 (sensitivity 0.85, specificity 0.80), respectively. A specificity of 0.7 combined with a sensitivity of 0.7 or higher was not reached by any ESAS-PHYS cut-off.

According to these scores, 62 (46.3%) had ESAS-TOT cut off of ≥ 20 (ESAS-TOT cases); of whom, 39 (62.9%) and 48 (77.4%) had a psychiatric diagnosis and DCPR diagnosis, respectively. Among ESAS-TOT cases, only 7 ICD-10 cases (15%) were excluded but 10 had a DCPR diagnosis without ICD-10 diagnosis. Fifty-six (41.8%) had ESAS-PSY cut off of ≥12 (ESAS-PSY cases), of whom, 39 (69.6%) and 44 (78.6%) had a psychiatric diagnosis and DCPR diagnosis, respectively. Among ESAS-PSY cases, only 7 ICD-10 cases (15%) were excluded but 7 had a DCPR diagnosis without ICD-10 diagnosis. Thirty-nine (84.8%) patients with a formal ICD-10 diagnosis did exceed both ESAS-TOT and ESAS-PSY cut-offs, while only 4 ICD-10 cases were not included in ESAS-TOT or ESAS-PSY cases.

### 3.3. ESAS Scores Among ICD-10, DCPR and CPC Cases

Mean and standard deviations on the ESAS for the different groups of patients, according to ICD-10 diagnoses are presented in Table 2. Higher scores on the ESAS symptoms (except shortness of breath and lack of appetite), ESAS-TOT, ESAS PHYS and PSY sub-score, as well as a higher number of problems on the CPC, were found among ICD-10 cases than ICD-10 no-cases (*p* < 0.05) (Table 2). Patients who received at least one DCPR diagnosis scored higher on ESAS psychological symptoms (depression, anxiety, feeling of not well-being), ESAS PSY and ESAS-TOT sub-scores, as well as on the total number on the CPC (Table 3). Patients were divided in three subgroups: those with an ICD-10 and DCPR diagnosis, those with a DCPR diagnosis but not an ICD-10 diagnosis and those not receiving any DCPR or ICD-10 diagnosis. Only three patients received an ICD-10 diagnosis but no DCPR diagnosis; due to the small size of this group, these patients were not considered for further analysis. Compared with patients who received at least one DCPR and no ICD-10 diagnosis, or those who received no diagnosis, patients who received both an ICD-10 diagnosis and a DCPR diagnoses showed higher scores on the ESAS symptoms (except for shortness of breath, lack of appetite, nausea, and pain), ESAS-TOT, ESAS PHYS and PSY sub-score, as well as a higher number of problems on the CPC. Compared with those with no ICD-10 or DCPR diagnosis, patients who received a DCPR diagnosis but no ICD-10 did not show any significant difference in the ESAS symptoms, ESAS-TOT, ESAS-PHYS or PSY sub-scores. The significant differences among these groups are shown in Table 4.

## 4. Discussion

To our knowledge, this is the first study in which ESAS has been reported as a valid, rapid, simple, easily understood screening instrument which allows to identify KTRs affected by psychiatric diagnosis.

Over 84.8% of ICD-10 cases exceeded both ESAS-TOT and ESAS-PSY cut-offs while only 4 ICD-10 cases did not have a total score superior to at least one of ESAS cut-offs. Our findings are in agreement with results reported in renal and haemodialysis patients [48], confirming ESAS as a reliable symptom assessment tool. In addition, regarding the intelligibility and clarity of ESAS, our KTRs reported that the instrument was easy to understand and simple to complete.

Regarding ESAS-PSY and ESAS-TOT scores, our results showed a modest tendency to overestimate caseness, applying an ESAS-PSY cut-off of ≥12 and an ESAS-TOT cut-off of ≥20. More specifically, ESAS-PSY and ESAS TOT cases were more than 18% to 26% compared to ICD-10 cases. However, 7 ESAS-PSY cases and 10 ESAS-TOT cases were DCPR cases without an ICD-10 diagnosis; and only 1 KTR had a total score superior to both ESAS-PSY and ESAS-TOT cut-offs without ICD-10 or DCPR diagnosis. With respect to this finding, ESAS was also able to recognize the presence of psychological dimensions requiring psychiatric care among ICD-10 no-cases, such as the quality of mood, the presence of sub-threshold anxiety or distress that accompanies conditions defined by DCPR.

As expected, no valid cut-off has been established for screening with ESAS-PHYS, although the value of ESAS-PHYS AUC indicated a moderate accuracy. In fact, these findings are consistent with data, indicating the sum of all 10-symptoms scores (ESAS-SDS), correlated more strongly with mental (*r* = −0.62, *p* < 0.001) rather than with physical (*r* = −0.54, *p* < 0.001) health in the renal population using KDQOL-SF [32].

The ESAS-psychological distress domain appeared to possess good psychometric properties to screen for caseness by assuming MINI6.0 as a ‘‘gold-standard’’ reference tool to assess psychiatric diagnosis in the KTRs. In fact, applying the ESAS-psychological distress with a cut-off point of ≥4 provided a good measure for screening caseness according to our study. This result is in line with other studies, which highlighted a cut-off score of ≥4 for detecting patients with emotional distress compared to scores on the HADS and the BSI-18 in cancer patients [48] and to scores on the Centre for Epidemiological Studies-Depression Scale (CES-D) in bone marrow transplant patients [53].

Furthermore, ESAS seemed to “balance” physical and psychological domains, including DT, and to better capture the emotional symptom burden. Indeed, all ESAS versions have physical symptoms outnumber items which induced patients to perceive the focus to be more physical than psychological symptoms [23]. The KTRs’ perception of emotional symptom burden is likely more important than any objective clinical parameters in determining the KTRs’ quality of life because KTRs reported many stressors, namely fear of rejection, worries about the risk of infection and prior to clinic visits, repeated hospitalizations, socio-economic status, side effects of immunosuppressive therapy and changes in body appearance [54].

Interesting results also emerged when examining the role of both DCPR and ICD-10 diagnoses on the single psychological and physical symptoms as measured by the ESAS and CPC problems. In fact, patients receiving either a DCPR or an ICD-10 diagnosis had higher levels of ESAS symptoms and problems in daily life. This result seems to indicate the role of psychosocial variables and distress in being associated with patients’ bodily and psychological feelings, with the possible negative influence on clinical outcomes also in KTRs (medication adherence, quality of life, morbidity, and mortality) [55,56].

The main limitation of our study is the small sample size of our population, which does not allow us to generalize our results. A future meta-analysis should be considered to overcome this important limitation and give more robust and generalizable results, although up to now, few studies on ESAS has been conducted in KTRs. Another limit is represented by the fact that, when conducting our study, the new version of the DCPR (DCPR-R) was not available yet.

In conclusion, this study provides information on the screening ability of ESAS to identify ICD-10 psychiatric diagnosis. ESAS could be a comprehensive and feasible screening instrument for symptom assessment in routine clinical practice among KTRs, easy to use by “non-specialists” in the topic under investigation, for an early referral of KTRs for psychiatric consultation. The implementation and regular use of the ESAS could guide clinical decision-making more easily than other psychiatric tools, which are not easy to interpret and often require many resources for data collection and analysis.

## Figures and Tables

**Table 1:** Socio-demographic and clinical variables of the sample.

Socio-Demographic Variables		Clinical Variables	
Age, years	56.13 ± 12	Previous psychological disorders	
Education, years	11.5 ± 4.52	Yes, *n* (%)	41 (30.6)
Sex		No, *n* (%)	93 (69.4)
Males, *n* (%)	90 (67.2)	Blood Test Values	
Females, *n* (%)	44 (32.8)	Hemoglobin, g/dL	12.4 ± 1.53
Marital Status		Calcemia, mmol/L	2.57 ± 1.2
Single, *n* (%)	29 (21.5)	Phosphoremia, mg/dL	3.27 ± 0.65
Married, *n* (%)	89 (66)	Total protein, g/dL	6.6 ± 0.71
Divorced, *n* (%)	10 (7.5)	Albumin, g/dL	5.8 ± 4.8
Widowed, *n* (%)	6 (4)	GFR-MDRD, mL/min	53.2 ± 17.5
Time after transplantation, median (IQR)	85(34.75–178.5)	BMI, kg/m^2^	24.5 ± 3.5
Smokers, *n* (%)	14 (10,4)	Systolic blood pressure, mm hg	130.4 ± 13.9
Living Situation		Diastolic blood pressure, mm hg	78.2 ± 7.9
Family, *n* (%)	93 (69.4)	Rank ICD diagnosis	
Parents, *n* (%)	22 (16.4)		
Alone, *n* (%)	11 (8.2)	No diagnosis, *n* (%)	88 (65.7)
Others, *n* (%)	8 (5.9)	Reaction to severe stress and adjustment disorders, *n* (%)	21 (15.7)
Occupation			
Employed, *n* (%)	49 (36.5)	Anxiety disorders, *n* (%)	14 (10.4)
Unemployed, *n* (%)	10 (7.4)	Mood [affective] disorders, *n* (%)	11 (8.2)
Retired, *n* (%)	59 (44.1)		
Housewives, *n* (%)	3 (2.2)		
Other, *n* (%)	13 (9.8)		

BMI: Body Mass Index; ICD: International Classification of Diseases; GFR-MDRD: glomerular filtration rate according to the equation from the Modification of Diet in Renal Disease Study.

**Table 2:** Mean (SD) scores on the ESAS and clinical characteristics among ICD-10 groups.

ESAS	ICD-10 Cases (*n* = 46)	ICD-10 Non-Cases (*n* = 88)	*p*-Value	CI 95% of the Difference
Pain	3.28 (±3.25)	2.07 (±2.99)	3 × 10^−2^ *	−2.32, −0.10
Tiredness	5.07 (±3.16)	3.01 (±2.82)	1 × 10^−3^ *	−3.11, −0.99
Nausea	1.22 (±2.32)	0.40 (±1.49)	3 × 10^−2^ *	−1.58, −0.06
Depression	3.78 (±2.59)	0.92 (±1.46)	1 × 10^−3^ *	−3.69, −2.04
Anxiety	5.96 (±2.69)	2.17 (±2.2)	1 × 10^−3^ *	−4.64, −2.93
Drowsiness	2.63 (±2.97)	1.31 (±2.08)	1 × 10^−3^ *	−2.31, −0.34
Lack of appetite	1.17 (±2.31)	0.48 (±1.46)	6 × 10^−2^ *	−1.45, 0.05
Feeling of not well-being	3.20 (±2.49)	1.24 (±1.9)	1 × 10^−3^ *	−2.79, −1.12
Shorten of breath	0.85 (±1.92)	0.88 (±1.95)	9.3 × 10^−1^ *	−0.67, 0.73
Distress	5.46 (±3.08)	2.41 (±2.28)	1 × 10^−3^ *	−4.08, −2.02
ESAS-PHYS	14.21 (±8.38)	8.13 (±7.66)	1 × 10^−3^ *	−8.93, −3.23
ESAS-PSY	18.39 (±7.62)	6.73 (±5.14)	1 × 10^−3^ *	−14.15, −9.16
ESAS-Total	32.6 (±13.36)	14.87 (±11.36)	1 × 10^−3^ *	−22.34, −13.13
CPC Total	4.72 (±2.27)	1.95 (±1.88)	1 × 10^−3^ *	−3.54, −1.98
Sex				
Males	*n* = 29 (32.3%)	*n* = 61 (67.8%)	4.6 × 10^−1^ ** (*X^2^* = 0.54, *df* = 1)	
Females	*n* = 17 (38.6%)	*n* = 27 (61.4%)		
Past psychopathology				
Positive history	*n* = 30 (26.8%)	*n* = 11 (73.2%)	1 × 10^−3^ ** (*X^2^* = 0.39, *df* = 1)	
Negative history	*n* = 16 (17.2%)	*n* = 77 (82.8%)		

CPC: Canadian Problem Checklist; DCPR: Diagnostic Criteria for Psychosomatic Research; ESAS: Edmonton Symptom Assessment System; ESAS-PHYS: physical distress sub-score; ESAS-PSY: psychological distress sub-score; ESAS-Total: global distress score; ICD−10: International Classification of Diseases–10th Revision; SD: Standard deviation. * Unpaired T-test. ** Chi-squared test.

**Table 3:** Mean (SD) scores on the ESAS and clinical characteristics among DCPR groups.

ESAS	DCPR Cases (*n* = 85)	DCPR Non-Cases (*n* = 49)	*p*-Value	CI 95% of the Difference
Pain	2.75 (±3.15)	2.02 (±3.05)	1.8 × 10^−1^ *	−1.83, 0.36
Tiredness	4.07 (±3.20)	3.10 (±2.82)	8 × 10^−2^ *	−2.05, 0.121
Nausea	0.73 (±1.86)	0.59 (±1.86)	6.8 × 10^−1^ *	−0.80, 0.52
Depression	2.45 (±2.52)	0.96 (±1.65)	1 × 10^−3^ *	−2.20, −0–77
Anxiety	4.09 (±3.01)	2.39 (±2.62)	1 × 10^−3^ *	−2.72, −0.68
Drowsiness	2.06 (±2.75)	1.24 (±1.88)	4 × 10^−2^ *	−1.61, −0.02
Lack of appetite	0.87 (±1.97)	0.45 (±1.50)	1.6 × 10^−1^ *	−1.02, 0.18
Feeling of not well-being	2.28 (±2.43)	1.27 (±1.93)	1 × 10^−3^ *	−1.77, −0.26
Shorten of breath	0.79 (±1.82)	1.00 (±2.13)	5.4 × 10^−1^ *	−0.48, 0.90
Distress	4.04 (±3.24)	2.45 (±2.05)	1 × 10^−2^ *	−2.49, −0.68
ESAS-PHYS	11.27 (±8.43)	8.40 (±8.12)	6 × 10^−2^ *	−5.79, 0.07
ESAS-PSY	12.85 (±8.89)	7.06 (±5.25)	1 × 10^−3^ *	−8.22, −3.38
ESAS-Total	24.13 (±15.40)	15.47 (±11.63)	1 × 10^−3^ *	−13.33, −3.99
CPC Total	3.45 (±2.49)	1.96 (±1.93)	1 × 10^−3^ *	−13.33, −3.99
Sex				
Males	*n* = 61 (67.8%)	*n* = 29 (32.2%)	1.3 × 10^−1^ ** (*X^2^* = 2.31, *df* = 1)	
Females	*n* = 24 (54.5%)	*n* = 20 (45.5%)		
Past psychopathology				
Positive history	*n* = 31 (75.6%)	*n* = 10 (24.4%)	5 × 10^−2^ ** (*X^2^* = 3.77, *df* = 1)	
Negative history	*n* = 54 (58.1%)	*n* = 39 (41.9%)		

CPC: Canadian Problem Checklist; DCPR: Diagnostic Criteria for Psychosomatic Research;.ESAS: Edmonton Symptom Assessment System; ESAS-PHYS: physical distress sub-score; ESAS-PSY: psychological distress sub-score; ESAS-Total: global distress score; SD: Standard deviation. * Unpaired T-test. ** Chi-squared test.

**Table 4:** Mean (SD) scores and significant differences on the ESAS among ICD-10 and DCPR groups (cases and no-cases).

ESAS	A. ICD-10 Cases DCPR Cases (*n* = 43)	B. ICD-10 No-Cases DCPR Cases (*n* = 42)	C. ICD-10 No-Cases DCPR No-Cases (*n* = 46)	ANOVA	Tukey’s Test *p*-Value (95%CI of the Difference)
Pain	3.19 (±3.21)	2.31 (±3.07)	1.85 (±2.94)	*F* = 1.92, *df* = 3, *p* = 1.2 × 10^−1^	-
Tiredness	5.19 (±2.98)	2.93 (±3.04)	3.09 (±3.21)	*F* = 5.25, *df* = 3, *p* = 1 × 10^−3^	A > B, *p* = 1 × 10^−3^ (−3.93, −0.59) A > C, *p* = 1 × 10^−3^ (0.47, 3.73)
Nausea	1.30 (±2.38)	0.14 (±0.78)	0.63 (±1.91)	*F* = 3.05, *df* = 3, *p* = 4.6 × 10^−1^	A > B, *p* = 2 × 10^−2^ (0.13, 2.19)
Depression	3.91 (±2.57)	0.95 (±1.32)	0.89 (±1.59)	*F* = 23.36, *df* = 3, *p* = 1 × 10^−3^	A > B, *p* = 1 × 10^−3^ (1.87, 4.04) A > C, *p* = 1 × 10^−3^ (1.96, 4.07)
Anxiety	6.07 (±2.61)	2.07 (±1.82)	2.26 (±2.51)	*F* = 25.86, *df* = 3, *p* = 1 × 10^−3^	A > B, *p* = 1 × 10^−3^ (2.65, 5.35) A > C, *p* = 1 × 10^−3^ (2.49, 5.13)
Drowsiness	2.79 (±3.01)	1.31 (±2.26)	1.30 (±1.93)	*F* = 3.98, *df* = 3, *p* = 1 × 10^−3^	A > B, *p* = 2 × 10^−2^ (0.12, 2.85) A > C, *p* = 2 × 10^−2^ (0.15, 2.82)
Lack of appetite	1.21 (±2.37)	0.52 (±1.40)	0.43 (±1.53)	*F* = 1.59, *df* = 3, *p* = 1.8 × 10^−1^	-
Feeling of not well-being	3.35 (±2.47)	1.19 (±1.85)	1.28 (±1.96)	*F* = 9.85, *df* = 3, *p* = 1 × 10^−3^	A > B, *p* = 1 × 10^−3^ (0.97, 3.35) A > C, *p* = 1 × 10^−3^ (0.90, 3.23)
Shorten of breath	0.91 (±1.97)	0.67 (±1.66)	1.07 (±2.18)	*F* = 0.51, *df* = 3, *p* = 6.3 × 10^−1^	-
Distress	5.70 (±3.02)	2.33 (±2.51)	2.48 (±2.08)	*F* = 16.40, *df* = 3, *p* = 1 × 10^−3^	A > B, *p* = 1 × 10^−3^ (1.93, 4.80) A > C, *p* = 1 × 10^−3^ (1.81, 4.63)
ESAS PHYS	14.58 (±8.35)	7.88 (±7.13)	8.36 (±8.19)	*F* = 6.40, *df* = 3, *p* = 1 × 10^−3^	A > B *p* = 1 × 10^−3^ (2.22, 11.18) A > C, *p* = 1 × 10^−3^ (1.83, 10.59)
ESAS PSY	19.02 (±7.26)	6.54 (±5.24)	6.91 (±5.09)	*F* = 40.70, *df* = 3, *p* = 1 × 10^−3^	A > B, *p* = 1 × 10^−3^ (9.10, 15.85) A > C, *p* = 1 × 10^−3^ (8.80, 15.41)
ESAS Total	33.60 (±12.91)	14.43 (±11.17)	15.28 (±11.63)	*F* = 23.70, *df* = 3, *p* = 1 × 10^−3^	A > B, *p* = 1 × 10^−3^ (12.42, 25.92) A > C, *p* = 1 × 10^−3^ (11.71, 24.92)
CPC Total	4.86 (±2.28)	2.00 (±1.78)	1.91 (±1.98)	*F* = 20.06, *df* = 3, *p* = 1 × 10^−3^	A > B, *p* = 1 × 10^−3^ (1.72, 4.00) A > C, *p* = 1 × 10^−3^ (1.84, 4.06)

ANOVA: Analysis of Variance; CPC: Canadian Problem Checklist; DCPR: Diagnostic Criteria for Psychosomatic Research; ESAS: Edmonton Symptom Assessment System; ESAS-PHYS: physical distress sub-score; ESAS-PSY: psychological distress sub-score; ESAS-Total: global distress score; ICD-10: International Classification of Diseases-10th Revision; SD: Standard deviation.

## Abbreviations

CPC	Canadian Problem Checklist
DCPR	Diagnostic Criteria for Psychosomatic Research
DSM	Diagnostic and Statistical Manual of Mental Disorders
DT	Distress Thermometer
ESAS	Edmonton Symptom Assessment System
ICD	International Classification of Diseases
KTRs	Kidney Transplant Recipients
MINI	Mini-International Neuropsychiatric Interview
